# Polymorphisms of the Steroid Sulfatase [*STS*] Gene are Associated With Attention Deficit Hyperactivity Disorder and Influence Brain Tissue mRNA Expression

**DOI:** 10.1002/ajmg.b.31120

**Published:** 2010-09-22

**Authors:** KJ Brookes, Z Hawi, J Park, S Scott, M Gill, L Kent

**Affiliations:** 1 Bute Medical School, University of St AndrewsSt Andrews, Scotland, UK; 2 Department of Psychiatry, Trinity Centre for Health Sciences, St James's HospitalDublin, Ireland

**Keywords:** steroid sulfatase, ADHD, association study, human brain tissue, mRNA

## Abstract

Previous studies in animals and humans have implicated the X-chromosome *STS* gene in the etiology of attentional difficulties and attention deficit hyperactivity disorder (ADHD). This family based association study has fine mapped a region of the STS gene across intron 1 and 2 previously associated with ADHD, in an extended sample of 450 ADHD probands and their parents. Significant association across this region is demonstrated individually with 7 of the 12 genotyped SNPs, as well as an allele specific haplotype of the 12 SNPs. The over transmitted risk allele of rs12861247 was also associated with reduced STS mRNA expression in normal human post-mortem frontal cortex brain tissue compared to the non-risk allele (*P* = 0.01). These results are consistent with the hypothesis arising from previous literature demonstrating that boys with deletions of the STS gene, and hence no STS protein are at a significantly increased risk of developing ADHD. Furthermore, this study has established the brain tissue transcript of STS, which except from adipose tissue, differs from that seen in all other tissues investigated. © 2010 Wiley-Liss, Inc.

## INTRODUCTION

Attention deficit hyperactivity disorder (ADHD) is one of the most heritable and prevalent childhood psychiatric disorders [Asherson, 2004], affecting an estimated 5% of school-aged children [Polanczyk et al., 2007]. Like most other neurobehavioral disorders, ADHD presents with a male bias [Swanson et al., 1998], which supports a possible role for X-linked genes in the etiology. Recent work from both human and animal studies suggests the X-linked gene; steroid sulfatase (*STS*) may have a role in attention. Boys with X-linked ichthyosis, who have a deletion or point mutation of *STS* are at an increased risk of ADHD [Kent et al., 2008] and common variants within the *STS* gene may increase susceptibility to ADHD [Brookes et al., 2008]. Both pharmacological manipulation of the steroid sulfatase axis and haplo-insufficiency of the *STS* gene have been shown to affect components of attentional functioning in mice [Davies et al., 2007, 2009]. STS, which escapes X inactivation in humans, desulfates several sulfated steroids including sulfated dehydroepiandrosterone, known as DHEA-S to DHEA. Both are neurosteroids with effects on several neurophysiological and behavioral processes including some evidence for an inverse relationship between DHEA blood levels and clinical symptomatology in boys with ADHD [Strous et al., 2001]. Additionally, methylphenidate, the commonest treatment for ADHD, has been shown to produce significant clinical improvement in boys with ADHD and simultaneous increases in serum levels of DHEA-S and DHEA, suggesting that these neurosteroids may play a role in the therapeutic effects of methylphenidate [Maayan et al., 2003].

We previously genotyped tagging SNPs (which capture the majority of diversity of genetic variation across a region) across the *STS* gene, with an evidence of association around intron 1 and 2 [Brookes et al., 2008]. This follow-up study, in an extended association sample has attempted to narrow down this region of association by genotyping more SNPs across intron 1 and 2. *STS* is known to have at least nine alternate first exons, with at least eight tissue specific transcripts [Dalla Valle et al., 2007; Zaichuk et al., 2007; Nardi et al., 2009], although brain tissue has not yet been fully investigated. We have investigated cDNA from human post-mortem brain tissue to establish the brain tissue transcript for *STS* and have investigated possible functional roles of associated SNPs through investigation of *STS* mRNA levels.

## MATERIALS AND METHODS

In this study, 450 probands and their parents were genotyped for 12 fine mapping STS SNPs. Eight of these SNPs had not been genotyped in our previous study of 383 probands while four of these SNPs (rs5934740, rs7058445, rs2270112, and rs12861247) had been previously genotyped in 383 of the 450 probands [Brookes et al., 2008]. Therefore the data for eight SNPs in all 450 probands is novel, and the data for the four previously genotyped SNPs is new for the additional 67 probands only.

### Association Sample

For this study 450 ADHD probands (inclusive of 12 affected siblings) and their parents were recruited from several child psychiatry clinics in the UK (N = 183) and Ireland (N = 267), following approval from the appropriate research ethics committees. Parental DNA was available for 98% of mothers and 68% of fathers. After complete description of the study to the subjects, written informed consent was obtained. For 380 of the probands from the UK and Ireland, parents were interviewed by trained psychiatrists or psychologists employing the Child and Adolescent Psychiatric Assessment (CAPA) [Angold et al., 1995]. Consistent interview procedures were employed across the two centers with researchers from each center receiving a common training in the use of the CAPA. Inter-rater reliability kappa coefficients were calculated for ADHD subtype diagnoses (κ = 0.82; CI = 0.71–0.94). The remaining 70 UK probands were interviewed with the parent version of the K-SADS interview [Kaufman et al., 1997] by a trained psychologist. Consistent interview procedures were employed across the two centers with researchers from each center receiving a common training in the use of the CAPA. Inter-rater reliability kappa coefficients were calculated for ADHD subtype diagnoses (κ =0.82; CI = 0.71–0.94). In addition, teacher ratings were obtained for children by the Conners Teacher Rating Scale (CTRS) [Conners, 1998]. This was to confirm that symptoms met the criterion of pervasiveness. Established cut-off points for possible and likely ADHD case ness on the CTRS were adhered to, that is, a *T*-score above 55 which was required. All 450 probands were White and born in the UK or Ireland (Age range 4–16 years). The sample was predominantly male (90.1%) with no significant difference in sex ratio between the two study recruitment centers. All probands fulfilled DSM-IV diagnostic criteria for ADHD. Of these N = 42 (9.3%) had the inattentive subtype, and N = 38 (8.4%) had the hyperactive impulsive subtype, the rest had combined subtype (82.3%). Children with an IQ below 70, autistic spectrum disorder or significant medical conditions such as epilepsy were excluded. Two-hundred eleven children (46.9%) had comorbid oppositional defiant disorder and 69 children (15.3%) fulfilled criteria for comorbid conduct disorder. Frequencies of subtype and comorbidity were similar across the two recruitment centers.

### Human Post-Mortem Brain Sample

Twenty-one (all male) samples of human post-mortem frontal cortex were obtained from the Medical Research Council (MRC) Sudden Death Brain and Tissue Bank, Edinburgh prior to this study. Deceased individuals were aged 16–70 years (mean = 46.3 years). RNA quality was measured by optical density (OD) with a Bioanalyzer (Agilent, CA). Sample OD_260_/OD_280_ ratios ranged from 1.5 to 2.0. Mean brain pH was 6.3 (SD = 0.25). The samples were crushed and stored at −80°C for future use. RNA and DNA extraction was carried out using the MELT™ Total Nucleic Acid Isolation System (Applied Biosystem™, Foster City, CA, 1983) and concentrations determined using a NanoVue (GE Health Care, UK). The cDNA was created with reverse transcriptase-mediated polymerase chain reaction (RT-PCR) using the AffinityScript™ QPCR cDNA Synthesis Kit (Stratagene, CA) on the Mx3005P machine (Stratagene).

### Association Study and Brain sample Genotyping

For association study samples, high molecular weight genomic DNA was extracted from whole blood, cheek swab or Oragene saliva collection systems (DNA Genotek, Ontario, Canada) according to routine procedures. Brain issue DNA was extracted as above. Fine mapping SNPs for *STS* were selected from Hapmap based on heterozygosity and linkage disequilibrium parameters and rs1473666 in exon 0b was identified as described below. SNP genotyping assays were obtained from Applied Biosystems for use on the Stratagene Mx3005P real-time PCR machine (Stratagene) following standardized protocols provided with the assays.

### Alternative First Exon Genotyping

The sequences of the recently documented six alternate first exons of *STS* designated 0a, 0b, 0c, 1a (exon 1a, 216 base pairs in size, is the previously known exon 1 of *STS*), 1b, 1c, and 1d by Dalla Valle et al. 2007, were compared to the human genome sequence using BLAST (http://blast.ncbi.nlm.nih.gov) to determine genomic location. Figure 4 demonstrates their relative positions. Exons 0a (351 bp) and 0b (77 bp) are in the 5′ region of the upstream gene, *5HDHD1A*, which is transcribed from the reverse strand, exon 0c (341 bp) is intragenic between *5HDHD1A* and *STS*, exon 1b (129 bp) is in the 5′ region of *STS* and exons 1c (98 bp) and 1d (246 bp) are within intron 1 of the *STS* gene. Two SNPs from dbSNP were identified, currently labeled as intronic, but which lie in these alternate first exons: both rs12839420 and rs1473666 reside in exon 0b but only rs1473666 was polymorphic.

Amplification of cDNA to establish the presence/absence of alternative first exons in the *STS* human brain transcript was performed on 21male and 7 female post-mortem brain samples. Primer pair amplifications were combined into two multiplex and two single plex reactions. Each PCR contained a positive control primer set for detecting cDNA in the known coding region of the *STS* gene, and a negative control pair set which detected intronic DNA (see Supplementary Table I). Genomic DNA was amplified in each reaction as a positive control for the presence of all alternative exons. cDNA samples were run in triplicate. Standard PCR conditions were employed and PCR products were visualized on a 2% agarose gel.

### DNA Sequencing

Mutation detection of STS exon 2 and the alternate exon 1's present in the brain transcript was performed by sequencing genomic DNA from 150 (for exon 2) and 100 (for exons 0a and 1b) male ADHD probands and 96 male controls obtained from Human Random Control DNA panels (HRC-1 and -2; European Collection of Cell Cultures, Sigma–Aldrich, Salisbury, UK). For exon 0a a product of 556 bp was obtained using primers (5′GCAGCTGTTCTCAAACGTTCACGC3′) and (5′GGCTCCGCGTCCTCTCCTCA3′); for exon 1b a product of 617 bp was obtained with primers (5′GGGTTGGGAACCCCTGCTGC3′) and (5′ACATGGTCTGGGTGAGTCAAACACT3′); for exon 2 a product of 713 bp was obtained using primers located within intron 1 (5′AGGCTGTTGACCCCATACAC3′) and intron 2 (5′GTGTCATGAAGGCAGGTGTG3′) and amplified using standard conditions and thermal cycles. Sequence was compared to genomic DNA NCBI ref sequence (NC_000023.10) using CodonCode Aligner version 3 (CodonCode Corporation) to identify novel polymorphisms.

cDNA PCR products from eight male human brain tissue samples covering the entire possible transcript from exon 0a in *5HDHD1a* through to the end of the 3′UTR of *STS* was also sequenced with 13 overlapping sequencing reactions. This was to confirm the agarose gel findings indicating the presence or absence of each alternate first exon in the brain transcript and to establish the entire transcript for STS in brain tissue. The forward primer of exon 0a combined with the reverse of exon 1b; and the forward primer of 1b and reverse primer of exon 2 were used for sequencing of the upstream region of the *STS* gene (see Supplementary Table I for sequences). Primer pairs (Supplementary Table II) were designed and used to sequence the rest of the coding sequence and transcribed 3′UTR sequences present in the mRNA. Standard PCR conditions were employed. All sequencing was performed commercially using an Applied Biosystems 3730 DNA sequencer by The Sequencing Service, University of Dundee. Sequence was compared to genomic DNA NCBI ref sequence (NC_000023.10) using CodonCode Aligner version 3 (CodonCode Corporation) to deduce transcribed regions in the brain transcripts.

### mRNA Expression

The *STS* primers and probe sequences were acquired from Applied Biosystems. The probe was FAM labeled and designed based on sequences spanning across exons 4 and 5 of the gene (Hs00996676_m1). Endogenous control gene assays used were human GAPDH and β-Actin (Applied Biosystems). Quantitative PCR was performed in triplicate for each sample on a Stratagene MX5003P using a standard protocol. The expression data produced were analyzed and converted into threshold cycle values (Ct values) using the software program MXPro Version 4.0 (Stratagene).

### Statistical Analyses

For the association studies, TDT analysis for each sample was carried out using the program software UNPHASED, which includes subjects with missing parental genotypes [Dudbridge, 2008]. In the current analysis a flag indicating the presence of an X-linked gene was included, therefore only maternal transmissions were counted. In addition, the UNPHASED program allows for permutations of the data, which allows some correction for multiple testing. Linkage disequilibrium across the SNPs and haplotype analysis were performed in Haploview [Barrett et al., 2005].

For the mRNA expression studies, mean values were obtained from the triplicate Ct values for each probe per sample. The target *STS* mRNA expression was normalized to two endogenous reference genes (GAPDH and β-Actin) to generate a ΔCt value. The relationship between the ΔCt values (*STS* mRNA expression) and the different genotype groups were analyzed by *t*-test in the analytical program SPSS Data Editor Version 17.0. In order to analyze both housekeeping genes together, geNORM software [Vandesompele et al., 2002] was used to create a gene expression normalization factor which is calculated for each sample based on the geometric mean of the housekeeping reference genes.

## RESULTS

### SNP Marker Association Study

**FIG. 1:**
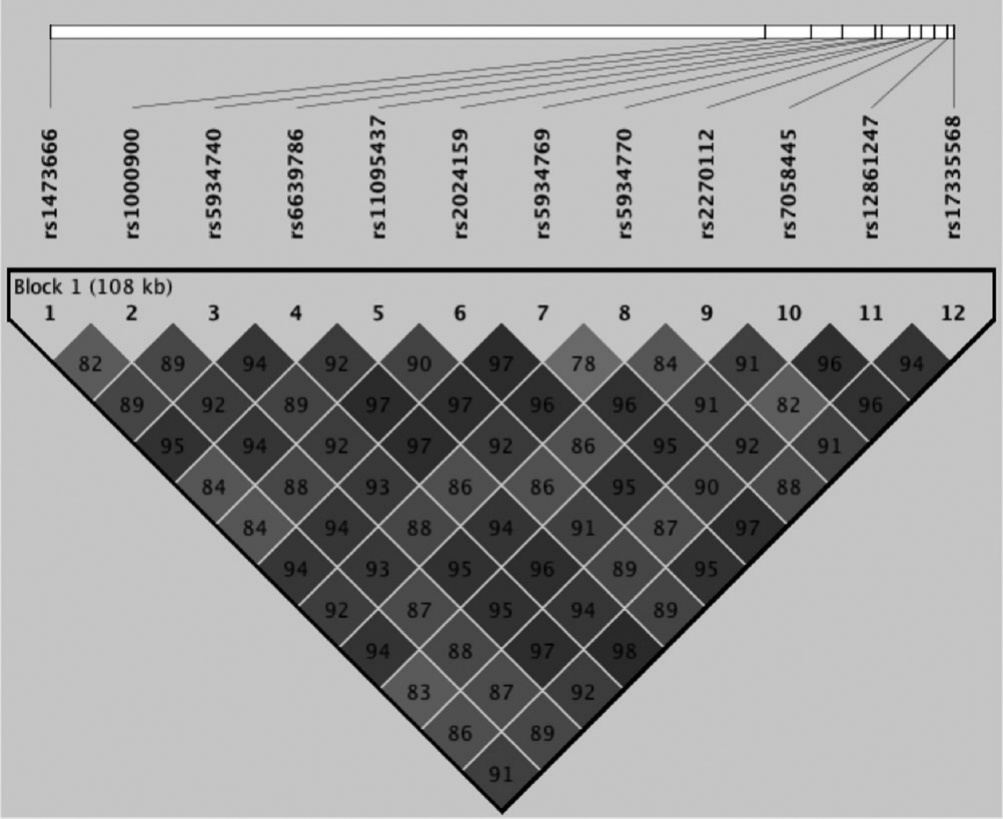
D′ linkage disequilibrium between STS SNPs.

Hardy–Weinberg equilibrium, calculated with allele frequencies from females only, was observed for all SNPs. Allele frequencies and genotype frequencies were similar between the two sites. Results are shown in Table I. Four tagging SNPs from the original study [Brookes et al., 2008] were also genotyped in this extended sample: rs5934740, rs7058445, rs2270112, and rs12861247. Of these, rs2270112 and rs12861247, which were significantly associated in the original study, were found to maintain significant association with ADHD in the extended dataset with similar odds ratios as the original study (rs2270112 OR was 1.48; rs12861247 was 2.05). Of the eight SNPs not previously genotyped, five SNPs residing in the first two introns also demonstrated significant association with ADHD. In the first intron of the *STS* gene three SNPs displayed significant association with ADHD: rs6639786 (*P* = 0.01), rs5934769 (*P* = 0.02), rs5934770 (*P* = 0.02). Within intron 2, one SNP indicated evidence of an association, rs1733558 (*P* = 0.01). In addition, rs1473666 in the 0b alternative exon also yielded a significant result (*P* = 0.05). All D′ linkage disequilibrium values are shown in Figure 1 which demonstrates one haploype block with low recombination. The application of the stringent multiple testing Bonferroni correction resulted in none of the SNP markers preserving statistical significance for association. An overall gene-wide significant test by permutation analysis (1,000 permutations) suggested a weak trend towards significance (*P* = 0.09).

**Table I:** Allele frequencies and TDT analysis result

SNP	Location	HWE	Genotyped	MAF	Informative trios	*P*-value	T	NT	OR	95% CI	Over-transmitted allele
rs1473666	Exon 0b	0.81	95.7	0.455	236	0.05	101	86	1.17	0.92–2.06	C
rs1000900	Intron 1	0.20	98.9	0.273	265	0.7	89	78	1.14	0.85–2.00	G
rs5934740	Intron1	0.78	91.5	0.461	211	0.33	87	81	1.07	0.75–1.77	G
rs6639786	Intron1	0.92	96.7	0.436	246	0.01	103	82	1.26	1.05–2.36	T
rs11095437	Intron1	0.05	99.1	0.233	266	0.36	85	69	1.23	0.97–2.36	T
rs2024159	Intron1	0.56	97.9	0.264	255	1	81	75	1.08	0.75–1.82	C
rs5934769	Intron1	0.13	86.9	0.423	224	0.03	83	69	1.20	0.92–2.26	T
rs5934770	Intron1	0.10	99.3	0.155	269	0.02	50	40	1.25	0.87–2.79	G
rs2270112	Intron 1	0.27	97.3	0.27	252	0.03	76	58	1.31	1.06–2.75	C
rs7058445	Intron 2	0.51	91.7	0.281	241	0.68	75	66	1.14	0.86–2.16	T
rs12861247	Intron 2	0.47	97.1	0.103	253	0.01	49	26	1.88	1.79–6.40	G
rs17335568	Intron 2	0.78	98.7	0.446	264	0.01	112	88	1.27	1.09–2.39	G

HWE, Hardy–Weinberg equilibrium; MAF, minor allele frequency; T, transmitted; NT, non-transmitted; OR, odds ratio; 95% CI, 95% confidence intervals.

Of all possible haplotypes, seven had at least a 1% frequency. There was significant over-transmission of one allele specific haplotype: CGGTTCTGCTGG (haplotype frequency = 0.23, χ^2^ = 10.13, *P* = 0.02), which consisted of all the significantly over-transmitted alleles for individual SNPs. This did not survive Bonferroni correction for the seven allele specific haplotypes tested.

The analysis was re-run excluding female probands (N = 47) to investigate the association in males. The markers rs12861247 (*P* = 0.02) and rs17335568 (*P* = 0.04) in intron 2 remained significantly associated and LD patterns across the region that did not alter substantially (see Supplementary Fig. 1 for LD plot of male only sample). It might be expected that ADHD females would have greater than expected homozygous rates for the significant risk alleles seen in hemizygous males. Therefore, the frequency of homozygote females for the risk alleles that were associated in the entire sample were compared to that estimated from the allele frequencies provided by HapMap or dbSNP. The sample was found to have a higher frequency of homozygous females for the risk G-allele of the marker rs5934770 than expected from estimations from dbSNP data (89% vs. 72%; *P* = 0.002). A trend for a higher frequency of females homozygous for the risk G-allele of rs12861247 (*P* = 0.06) was also observed. Neither of the SNPs associated with the male only analysis had significantly higher than expected risk allele homozygotes in the females.

### Alternate First Exon Usage in Brain Transcript

**FIG. 2:**
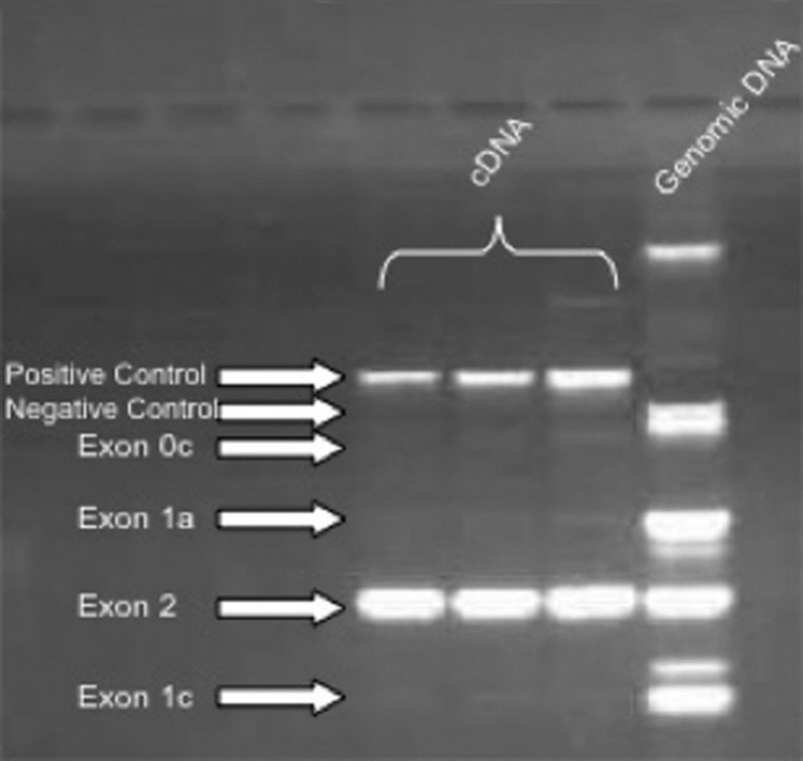
PCR results for the presence in brain tissue of alternative exons 0c, 1a, 1c, and 2.

**FIG. 3:**
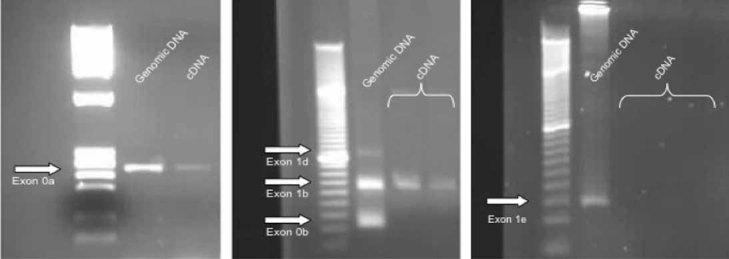
PCR results for the presence in brain tissue of alternative exons 0a, 0b, 1b, 1d, and 1e.

Figures 2 and 3 demonstrate all alternative first exons present in the cDNA with the negative control for mRNA (the amplification of intron 2 sequence which should only be present in genomic) and the positive control for mRNA (the sequence spanning exons 3–5 which is too large to resolve from genomic) in Figure 2. Within the brain *STS* transcript exons 0a, 1b, and 2 are the most abundant exons present, with no apparent detection of exons 0b, 0c, 1a (exon1), 1c, 1d, and 1e. Exon 0d was too small to amplify (17 bp) but was not present in the DNA sequencing (see below).

### DNA Sequencing

**FIG. 4:**
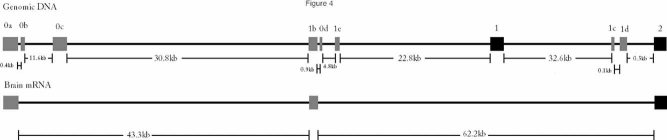
Schematic diagrams showing alternate first exons present in brain transcript mRNA in relation to genomic DNA.

Sequencing of exon 0a, 1b, exon 2 in genomic DNA of 150 male ADHD probands and 96 male controls revealed no new polymorphisms. Sequencing of the *STS* gene cDNA, created from RNA isolated from 8 of the 21 post-mortem brain samples confirmed the inclusion of the alternative exons 0a and 1b [as described by Dalla Valle et al., 2007] and exon 2 in the transcript (schematic in Fig. 4). No new polymorphisms were detected in the coding sequence or the UTRs present in the transcript. From exon 2 downstream, the transcript was identical to NCBI reference sequence (NM_000351).

### STS mRNA Expression

**FIG. 5:**
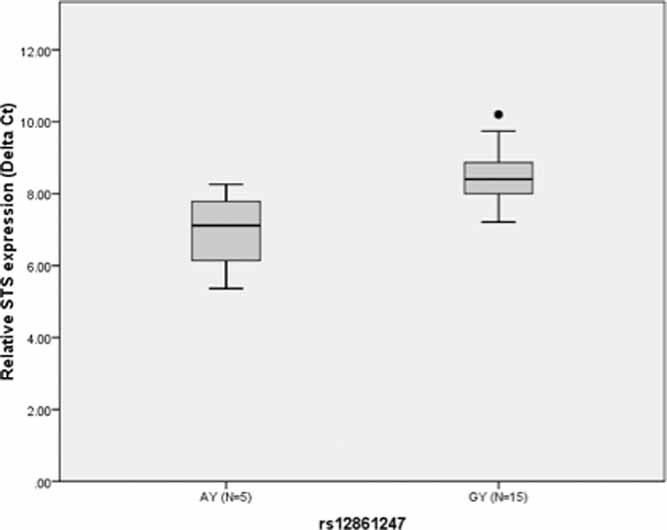
Relative STS expression compared to GAPDH for rs12861247. Higher values correspond to lower STS mRNA levels. Error bars represent 95% CI.

Gene expression of the *STS* gene in human post-mortem frontal cortex samples was approximated by the measurement of mRNA levels via quantitative PCR. Expression data in relation to the seven SNP markers that were observed to show statistical significance for association with ADHD were analyzed. Relative *STS* mRNA expression levels were normally distributed by Kolmogorov–Smirnov tests, and co-factors such as post-mortem interval, age, sex, and pH were not correlated with *STS* mRNA or protein expression level and therefore not included as covariates. There were no batch effects for different Quantitative-PCR runs. A significant relationship between the G-allele of rs12861247 and lower *STS* mRNA expression relative to GAPDH was noted (*t* = −2.76, *P* = 0.01; Fig. 5). This does not survive Bonferroni correction for the seven SNPs tested. No associations with *STS* mRNA expression were evident for any SNP when both housekeeping genes were analyzed together using GeNORM (see Supplementary Fig. 2).

## DISCUSSION

In this study, we have demonstrated further evidence for association of the *STS* gene with ADHD. This study focussed on findings from previous work [Brookes et al., 2008] by initially fine mapping a region spanning intron 1 and 2. Novel findings for five SNPs in this region were found with significant evidence for association: rs6639786, rs5934769, rs5934770 located within intron 1; rs17335568 located within intron 2; and rs1473666 in the alternate exon (0b) located upstream in the 5′ region of *5HD1A1A*. Two of the previously genotyped SNPs, rs2270112 and rs12861247 were also still significant in this larger sample. However, none survived stringent correction for multiple testing and 1,000 permutations of the data provided a trend for significance only. Linkage disequilibrium between the individual SNPs is very high with all the new associations in this study being in high LD with the four SNPs significantly associated in our previous study [Brookes et al., 2008]. At this stage it is therefore difficult to determine which SNP, or SNPs are responsible for the significant association, and which appear significant by virtue of LD with other SNPs. Further studies to determine which SNPs have functional effects on gene expression and on downstream targets such as STS activity and DHEA /DHEA-S levels may provide some indirect evidence for which SNP/s are relevant.

Of the seven significantly associated SNPs, risk alleles for four (rs5934770, rs2270112, rs12861247, rs17335568) are predicted to create new transcription factor binding sites (http://www.cbrc.jp/research/db/TFSEARCH.html). It is well established that transcriptional regulatory elements can be located within introns, particularly intron 1 [e.g., Surinya et al., 1998; Gemel et al., 1999; Ozaki et al., 2002]. Several binding sites in the Rel/NFKappa transcription factor family are created by the risk allele for rs17335568 and a upstream transcription factor 1 binding site for the risk allele of rs5934770, a transcription factor involved in lipid and glucose homeostasis, although it is not immediately apparent how these particular binding sites might be involved in ADHD pathophysiology. Of more interest is the POU3F1/Tst-1/Oct 6 binding site, predicted to be abolished by the risk allele of rs12861247, which may impact on mRNA expression. POU3F1 is a member of the POU-III family of homeodomain proteins, whose primary role is thought to be developmental. It is expressed in cells of the oligodendrocyte lineage [Collarini et al., 1992; Wegner, 2001] and Frantz et al. 1994 suggested it also played a role in regulating transcription in differentiated CNS neurons as well as in proliferating glial precursors.

Of particular interest is the finding that affected females, compared to Hapmap or dbSNP allele frequencies were statistically more likely to be homozygous for the G-risk allele of rs5934770 and showed a trend for more homozygotes for the G-allele of rs12861247. In our previous study [Brookes et al., 2008], we found statistically more homozygote females for the G-allele of rs12861247 and hypothesized a model whereby females would need to be homozygote for *STS* gene risk alleles in order to increase their susceptibility to ADHD. These two SNPs located in intron 1 and 2, respectively have high linkage disequilibrium between them (D′ = 0.92) and may not be independent findings. They may also be “tagging” another polymorphism, but if so, this is unlikely to be a common polymorphism in exon 2 as sequencing in 150 ADHD male probands and nearly 100 controls did not reveal any new mutations. Therefore, further investigation of the association findings were focussed upstream of exon 2.

Tissue specificity of STS transcripts has been recently reported [Dalla Valle et al., 2007; Zaichuk et al., 2007; Nardi et al., 2009], although brain tissue specificity has not been widely investigated. The presence or absence of each of the possible seven alternate first exons originally described by Dalla Valle et al. 2007 was investigated by RT-PCR in human post-mortem brain tissue using individual primer pairs for each exon and confirmed by sequencing of the entire transcript. PCR products could be amplified in the brain transcript for exons 0a, 1b, and 2. This is identical to one of the two tissue specific transcript for adipose tissue identified by Dalla Valle et al. 2006. This transcript does not appear to contain exon 1a, otherwise known as exon 1, which is in the placental transcript. Zaichuk et al. 2007 demonstrated that transcripts containing exon 1b were by far the most abundant in brain tissue (6,107 amol/mg) but they also detected minute amounts of exons 1a (7.6 amol/mg) and 1c (2.0 amol/mg), but at such small levels of detection these could arguably be as a result of measurement error. It is possible that other exons are present in brain tissue but as RT-PCR will favor amplification of the most abundant transcript, the quantity produced may not have been detectable by our methods.

Exon 1b is highly homologous to several non-human primate STS mRNAs, suggesting a preserved functional role. It contains a putative ATG start site, with a stronger Kozac consensus sequence than the in frame ATG start site in exon 2. Use of the exon 1b start site would result in a transcript 12 amino acids longer at the N-terminal than that produced from the exon 2 start site, and seven amino acids longer than that produced in placental tissue that uses the exon 1 (exon 1a) start site. Signal peptide prediction (http://www.cbs.dtu.dk/services/SignalP/) suggests that this difference has no impact on the signal peptide cleavage site. The presence of exon 0a upstream of exon 1b in the brain transcript is a novel finding in this study although this exon is not translated [Dalla Valle et al., 2007; Nardi et al., 2009]. Additionally, although there is a significant association of rs1473666 in the alternate exon 0b with ADHD this is more likely to be as a result of this variant being in LD with another significantly associated marker, than a true functional variant, as this exon does not appear to be present in the brain transcript, and is not translated in tissue transcripts that do contain it [Dalla Valle et al., 2006; Nardi et al., 2009]. Given the presence of exon 0a and 1b in the brain transcript, we also sequenced genomic DNA for these exons in 150 ADHD male probands and nearly 100 controls but did not discover any new polymorphisms.

Levels of STS mRNA were also investigated in human post-mortem brain tissue in relation to genotype. We hypothesized that because boys with STS deletions, and therefore no detectable STS protein, have higher than expected rates of inattentive ADHD, some boys with ADHD would be predicted to have lower levels of STS mRNA. Although the G-allele of rs12861247, which was significantly associated with ADHD, was also significantly associated with a lower STS mRNA level relative to the GAPDH control gene, using an alternative method of analysis, with more than one house keeping gene control, did not support this initial finding. However, brain tissue is composed of a variety of cell types such that mRNA expression levels of genes may vary between samples as a result of differences in cell composition of the particular piece of tissue used. These findings for STS mRNA expression levels in relation to genotype in brain tissue therefore require further investigation, preferably using a more cell specific approach such as laser capture micro-dissection techniques. The use of post-mortem brain tissue still has several advantages over in vitro techniques. The endogenous promoters and all other gene sequences, etc. needed for gene regulation are present in the tissue and this arguably provides a more relevant paradigm than cell lines and reporter vector systems, which do not represent normal tissue. However, Additionally, the tissue employed for this study was from “normal” individuals with no known history of psychiatric disorder (although this cannot be ruled out) and the sample size was small, and possibly limited in power to detect what are likely to be small effect sizes of these SNPs. Additionally, the small sample size may not allow the detection of rare brain transcripts that use exons 1a and 1c as suggested by Zaichuk et al. 2007.

In summary, this association study provides further evidence that genetic variation in the STS SNPs (rs1286124 gene may be implicated in ADHD susceptibility. One of the significantly associated 7) in intron 2 may be associated with lower STS mRNA expression as well as being more prevalent in female ADHD homozygotes. Replication of these findings is needed as well as further functional investigation of the associated alleles.
