# Supplementary figures and images for: Polymorphisms of the Steroid Sulfatase [STS] Gene are Associated With Attention Deficit Hyperactivity Disorder and Influence Brain Tissue mRNA Expression
Source: Am J Med Genet B Neuropsychiatr Genet. 2010 Sep 22;153B(8):1417–24. doi: 10.1002/ajmg.b.31120 (PMC3132592; doi:10.1002/ajmg.b.31120)

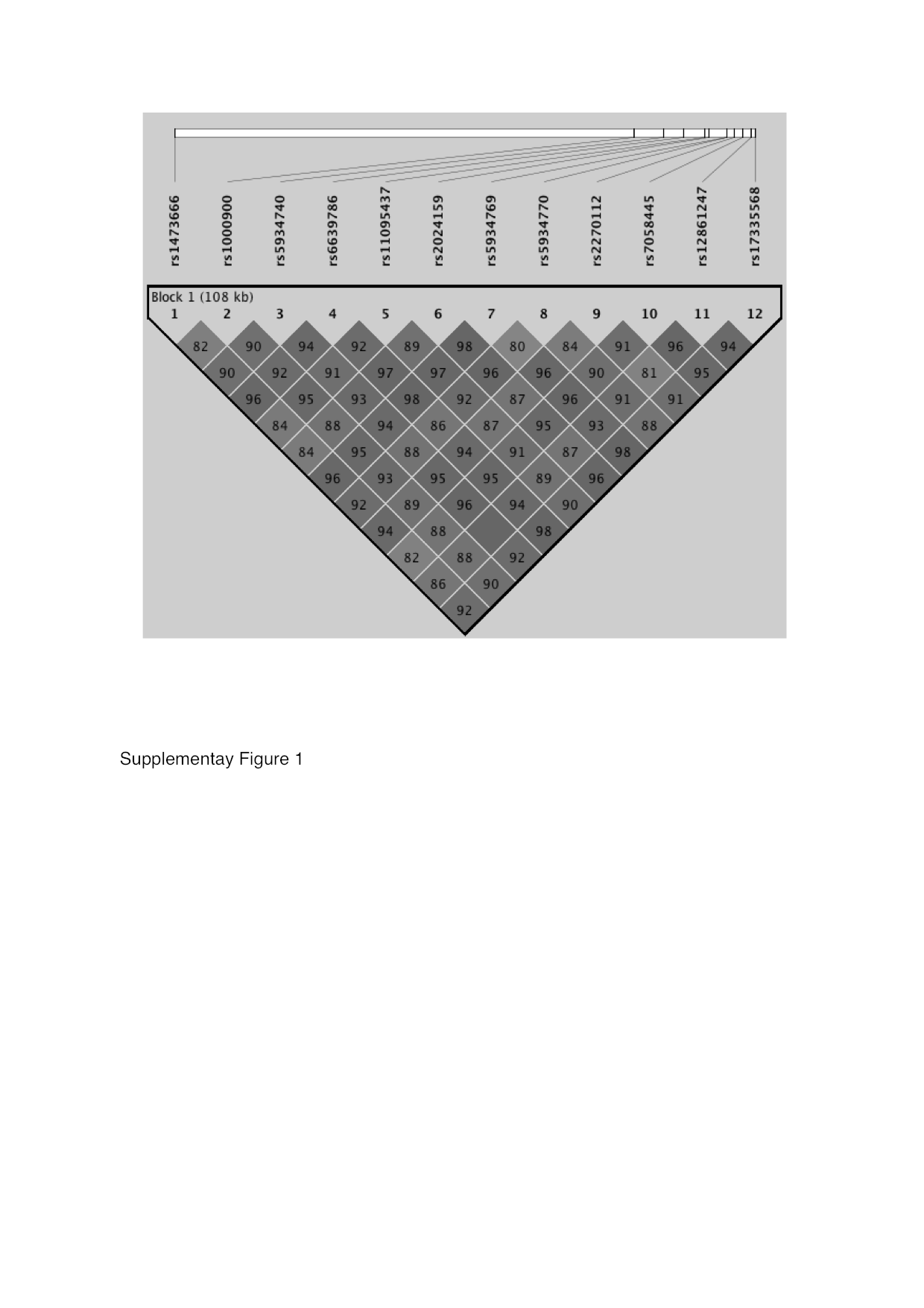

Supplement: Supplementary file 1 [file ajmg153B-1417-SD1.tif]

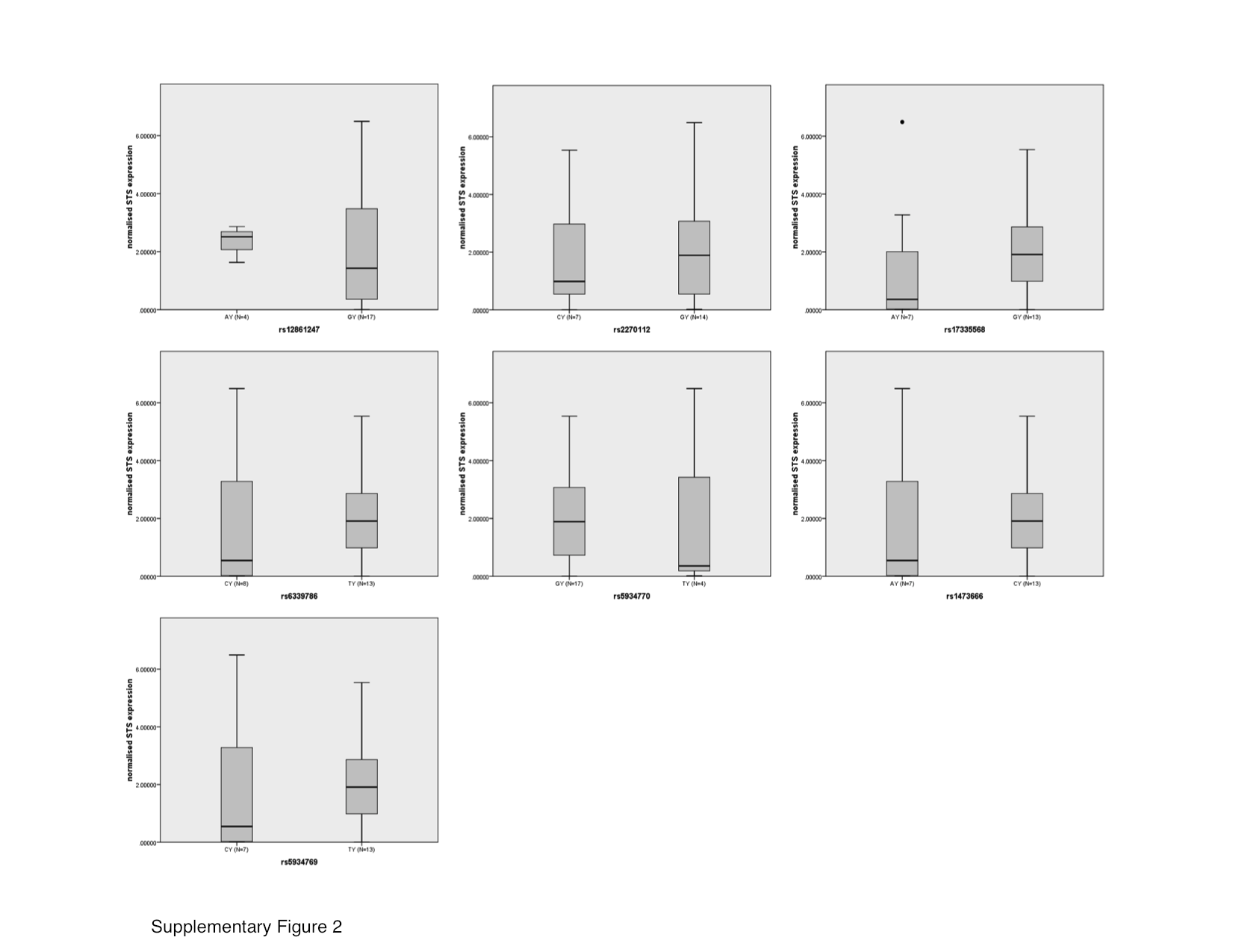

Supplement: Supplementary file 2 [file ajmg153B-1417-SD2.tif]
