# Supplementary material for: Polymorphisms of the Steroid Sulfatase [STS] Gene are Associated With Attention Deficit Hyperactivity Disorder and Influence Brain Tissue mRNA Expression
Source: Am J Med Genet B Neuropsychiatr Genet. 2010 Sep 22;153B(8):1417–24. doi: 10.1002/ajmg.b.31120 (PMC3132592; doi:10.1002/ajmg.b.31120)
Supplement: Supplementary file 3 [file ajmg153B-1417-SD3.doc]

### Supplementary Table 1: Alternative First Exon Genotyping primers

| **Primers** | **Sequence (5’ – 3’)** | **Product Length**  **(bp)** |
| --- | --- | --- |
| **Exon 0a**  Forward  Reverse | CGGACCTTCTGTGCCTCTAC  CTCCAAGGTTGCTGCTGTG | 273 |
| **Exon 0b**  Forward  Reverse | GCACACTACCCACCCAGAAG  TCATCTTTGACATGGACGGA | 43 |
| **Exon 0c**  Forward  Reverse | TGGTGAAGGAAGTCCAGCTT  TCGGGCTCTTCCAGTTCTTA | 336 |
| **Exon 1a**  Forward  Reverse | CTGTTGGCCAAGCCTCC  TCCAGCTTGTGATCCTGTTG | 213 |
| **Exon 1b**  Forward  Reverse | GATGCCCTTGGTTTGACTCT  AAGACGATCTTGCGCCATT | 106 |
| **Exon 1c**  Forward  Reverse | TCAAGGCTGTGAACCAGTTG  CCTTCTAGTCTTCCCACCAGC | 65 |
| **Exon 1d**  Forward  Reverse | GGCAGCAAAACCTCAAGAGT  GAGGATGGTGGCTTGAACAC | 169 |
| **Exon 2**  Forward  Reverse | AAGATGAAGATCCCTTTCCTCC  GTTTTGTTCCCATAGCACCC | 134 |
| **Positive control**  **(Exon 3-5)**  Forward  Reverse | GATCAGGAATGGCATCTTGG  ATCATGAAGCAGTTCAGGGG | 466 |
| **Negative control**  **(Intron 2)**  Forward  Reverse | ATGCAAGGGTAAGATCTGCAA  ATGAATTCTTCCATGGGGCT | 362 |
