# Supplementary material for: Polymorphisms of the Steroid Sulfatase [STS] Gene are Associated With Attention Deficit Hyperactivity Disorder and Influence Brain Tissue mRNA Expression
Source: Am J Med Genet B Neuropsychiatr Genet. 2010 Sep 22;153B(8):1417–24. doi: 10.1002/ajmg.b.31120 (PMC3132592; doi:10.1002/ajmg.b.31120)
Supplement: Supplementary file 4 [file ajmg153B-1417-SD4.doc]

Supplementary Table 2: Brain transcript sequencing primers

| ***Primers*** | ***Sequence ( 5’-3’)*** | ***Product Length (bp)*** |
| --- | --- | --- |
| **Coding1**  Forward  Reverse | TTGGAGATCCTGGGTGCTAT  ATCATGAAGCAGTTCAGGGG | 619 |
| **Coding2**  Forward  Reverse | CCTTTTCTTGGGCTTCCTTC  GTTGAAGTTGGGGGTGAAGA | 739 |
| **Coding3**  Forward  Reverse | CTTGAAGGAAAAAGCCAACG  CCCAAGCCTTCAGGTGATAA | 609 |
| **UTR1**  Forward  Reverse | ACTTTCTTTGGAAGCCCTGG  TGCCTTCCAATAACCAAAGTG | 661 |
| **UTR2**  Forward  Reverse | GCAGTGATGCAATAACCAGC  GCGCATTTTCCCTGATTTAC | 763 |
| **UTR3**  Forward  Reverse | TGCTTCCAATCCACAATGAG  ATCCCACAATTTCTGGAACG | 702 |
| **UTR4**  Forward  Reverse | TTCCTACCCCCTACCTCCAG  TGGATATGCAACTCCACAGG | 715 |
| **UTR5**  Forward  Reverse | GTAAGGCATGGCTGCTTGTT  TAATAATCGGGCCTTTTCCC | 666 |
| **UTR6**  Forward  Reverse | GGCTGAGAAATGCAGAGAAGA  AATGAGAGCCGATCCTAGTCC | 510 |
| **UTR7**  Forward  Reverse | CTAGGATCGGCTCTCATTCG  TGCATCACACATCCCATTCT | 194 |
| **UTR8**  Forward  Reverse | AATGGGATGTGTGATGCAAA  AGCAACACTGGCTTGAGTGA | 707 |
